# Can urban gardens improve food security, health, well-being and financial sustainability of households?

**DOI:** 10.1080/07853890.2021.1896077

**Published:** 2021-09-28

**Authors:** Helena Guerreiro, Tânia Fernandes, Madalena Bettencourt da Câmara

**Affiliations:** a Instituto Universitário Egas Moniz (IUEM), Egas Moniz Cooperativa de Ensino Superior, Caparica, Portugal; b Centro de Investigação Interdisciplinar Egas Moniz (CiiEM), Egas Moniz Cooperativa de Ensino Superior, Caparica, Portugal

## Abstract

**Introduction:**

An increased consumption of fresh fruit and vegetables is associated with health benefits [1,2]. Including them in the daily diet can reduce the risk of noncommunicable diseases [2]. Urban gardens (UG) can improve communities around them by allowing a supply of such products [3,4], by contributing to conscious decisions about eating [4] and lessening the health costs of its populations [2–4], by improving their participants self-esteem, and by helping in the development of competencies [4]. This preliminary study aimed to understand the parameters that could be evaluated on a greater scale future study, to assess the role of urban gardens in their participants health, nutrition knowledge and family budget.

**Materials and methods:**

An UG in Setúbal, Portugal, was selected for this study. All data was collected in October, 2018 and included: demographic data from the gardeners (*n* = 133), a “Food and Nutrition Knowledge” validated questionnaire [5] with 20 true and false questions (*n* = 6), and semi-structured interviews (*n* = 6). All interviewed gardeners understood the purpose and signed the informed consent.

**Results:**

The UG had 138 plots with 133 in use. Most of the gardeners were men (59.9%) and under 65 years of age (74.4%). 30,5% had an income under (*n* = 133) 11,999€per year.

**Discussion and conclusions:**

Our results meet those of recent studies that enhance the importance of UG in feeding populations, especially those with diminished food security and low access to fresh quality products [6–8]. Increased level of activity, higher fruit and vegetable consumption, along with savings in the family budget were referred by gardeners supporting the need for a more in-depth study. The UG is eligible for an observational study using the methods already stated. In addition, collection of anthropometric data and nutritional and cognitive screening tests would allow for a better view of the gardeners health.
